# A Therapeutic Role for Survivin in Mitigating the Harmful Effects of Ionizing Radiation

**DOI:** 10.1155/2016/1830849

**Published:** 2016-04-17

**Authors:** Katherine H. Carruthers, Gregory Metzger, Eugene Choi, Matthew J. During, Ergun Kocak

**Affiliations:** ^1^The University of Toledo College of Medicine, 3000 Arlington Avenue, Toledo, OH 43614, USA; ^2^The Ohio State University Department of Plastic Surgery, 915 Olentangy River Road, Suite 2100, Columbus, OH 43212, USA; ^3^The Ohio State University Department of Molecular Virology, Immunology, and Medical Genetics, 370 West 9th Avenue, Columbus, OH 43210, USA; ^4^Midwest Breast and Aesthetic Surgery, 1329 Cherry Way Drive, Suite 200, Gahanna, OH 43230, USA

## Abstract

*Background*. Radiation therapy is a form of adjuvant care used in many oncological treatment protocols. However, nonmalignant neighboring tissues are harmed as a result of this treatment. Therefore, the goal of this study was to induce the production of survivin, an antiapoptotic protein, to determine if this protein could provide protection to noncancerous cells during radiation exposure.* Methods*. Using a murine model, a recombinant adenoassociated virus (rAAV) was used to deliver survivin to the treatment group and yellow fluorescence protein (YFP) to the control group. Both groups received targeted radiation. Visual inspection, gait analysis, and tissue histology were used to determine the extent of damage caused by the radiation.* Results*. The YFP group demonstrated ulceration of the irradiated area while the survivin treated mice exhibited only hair loss. Histology showed that the YFP treated mice experienced dermal thickening, as well as an increase in collagen that was not present in the survivin treated mice. Gait analysis demonstrated a difference between the two groups, with the YFP mice averaging a lower speed.* Conclusions*. The use of gene-modification to induce survivin expression in normal tissues allows for the protection of nontarget areas from the negative side effects normally associated with ionizing radiation.

## 1. Introduction

Radiation therapy is a common form of adjuvant care used in many oncological treatment protocols following surgical resection of a mass lesion. It is especially important for the treatment of tumors associated with a high rate of local recurrence, such as Ewing sarcoma, to design a plan that combines both surgical resection of the primary lesion and pre- and/or postoperative radiation therapy to a targeted region in order to treat remaining microscopic disease [1–4]. Ionizing radiation can be very effective in disrupting the growth of any remaining malignant cells; however, nonmalignant neighboring cells are inevitably affected as well. It is widely acknowledged that even limited radiation exposure to surrounding tissues can cause significant delays in wound healing and can ultimately lead to the breakdown of even previously healed reconstructions. Most of these negative effects on wound healing stem from DNA damage, increased inflammation, and cell apoptosis, which ultimately compromise the body's ability for self-repair [5, 6]. Wound breakdown due to radiation exposure can limit radiation dosing and dramatically hinder the patient's rehabilitation process [7, 8].

It is these negative effects that cause radiation treatment to be viewed with apprehension, despite its oncological benefits, and research has long been focused on finding methods to reduce the unintentional damage to tissues surrounding the target radiation site [9–16]. Publications have emerged that suggest the use of topical* Aloe vera* gel, beclomethasone spray, or hyperbaric oxygen as possible modes to reduce both acute and long-term radiation-induced injury to normal local tissues [17–19]. Although these techniques represent large advances, they are limited by their duration of effectiveness. The proposed treatment regimens must be readministered with every subsequent exposure to radiation and the systemic effects of these treatments have not been well established. Therefore, to date, no single technique has been shown to be locally effective in the long-term prevention of radiation-induced tissue injury and, thus, acceptance of collateral damage and management of the negative side effects associated with radiation therapy remain the mainstay of treatment.

Gene-modification has been employed for many years to introduce nonnative proteins to the human body for therapeutic purposes. The protein survivin has been found in elevated levels in human cancer cells and has been shown to directly correlate with the cellular growth and proliferation phases [20]. It is speculated that the role of survivin may be to decrease the cell's susceptibility to apoptosis and help malignant cells avoid the body's natural defenses that would normally lead to cell death. In contrast, healthy, nonmalignant human tissue does not normally express measurable levels of survivin [21–23].

The purpose of this study was to use gene-modification techniques previously described by our laboratory to induce the production of the protein, survivin, in tissues that would subsequently be exposed to therapeutic doses of radiation to determine if this unique protein could prove protective against the harmful effects of radiation treatment on noncancerous cells.

## 2. Materials and Methods

### 2.1. Murine Model

Eight-week-old immunocompetent C57BL/6 mice or CD-1 IGS mice (Charles River Laboratories, Wilmington, MA) (approximate weight 22 g) were randomly divided into treatment and control groups with five mice in each population. All mice were treated in accordance with the guidelines approved by The Ohio State University Institutional Animal Care and Use Committee (IACUC) (approval #2010A00000084).

### 2.2. Viral Vector Construction

A recombinant adenoassociated virus (rAAV) was used for local delivery of the chosen protein to the left hind leg of each animal. This rAAV vector, known as serotype rAAVrec2, was derived in our laboratory using a PCR shuffling technique from human and novel nonhuman primate viral isolates and has been successfully employed in other gene therapy protocols, including multiple studies previously published by our laboratory [24, 25]. Specifically, a rAAV vector containing the gene for either murine-sourced survivin or yellow fluorescent protein (YFP) was constructed. The cDNA was cloned into the high expression pAM AAV cis-plasmid containing the hybrid CBA promoter and WPRE 3′ sequence. The subsequent pAAV-CBA-WPRE was used to generate high titer rAAV vectors expressing either survivin or YFP using transfection techniques with helper plasmids as previously described by our laboratory. The resulting rAAV-survivin vector was used in the treatment group and rAAV-YFP vector was used as a marker in the control group.

### 2.3. Gene Therapy Administration

Gene-modification was accomplished using direct injection of the viral vector using a 50 *μ*L Hamilton syringe with a 30-gauge needle. Previous experiments conducted in our laboratory have identified this as an effective method for localizing the viral vector gene products while limiting the operative time required for transduction [24, 25]. Viral vectors were titered using real-time PCR. Stock solutions of rAAV-survivin and rAAV-YFP each were diluted to a concentration 6.7 × 10^11^ vg/mL. From that stock solution, treated mice received 1 × 10^10^ virions of rAAV-survivin in 15 *μ*L and the control mice received 1 × 10^10^ virions of rAAV-YFP in 15 *μ*L via intramuscular injection in the dorsal portion of the left quadriceps femoris muscle.

### 2.4. Adjuvant Radiation Model

**Figure 1:**
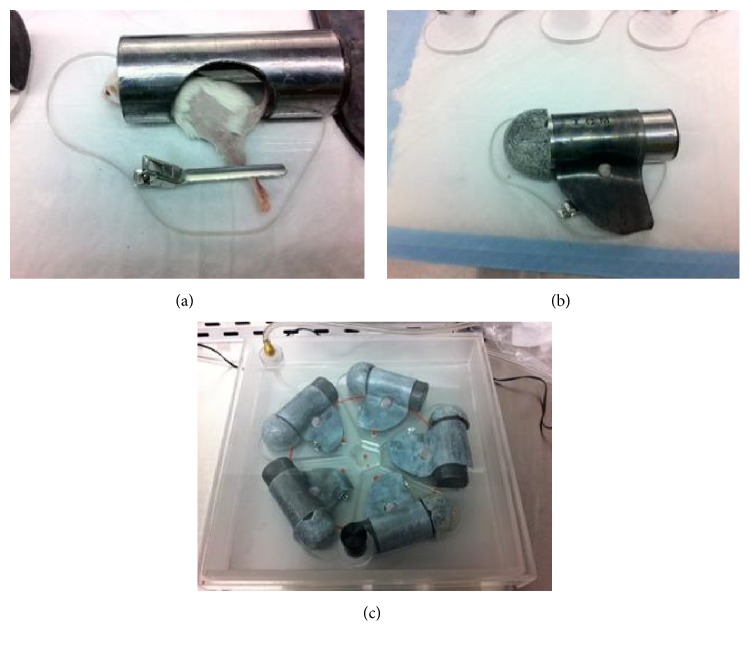
Fractionated radiation therapy was selectively administered to each animal. A specialized jig was designed and built out of lead in order to ensure that only the gene-modified dorsal aspect of the left hind leg was exposed to the radiation beam (a, b). Five animals at a time were anesthetized and placed in a radiation chamber where they were exposed to 5 Grays of radiation at a dosage rate of 1 Gy/min (c). This protocol was carried out for ten consecutive days for a cumulative dose of 50 Gy/animal.

One week following administration of the viral vector, all animals received their first dose of radiation. Mice were anesthetized via intraperitoneal injection of ketamine/xylazine (87/13 mg/kg) and the left hind leg was shaved and sterilized using alcohol prep. Using the same unique lead shield and protocol employed by Lu et al., targeted radiation therapy was applied to the dorsal aspect of the left leg, where the mice had previously received their gene-modification [26]. Five animals were irradiated at the same time, each housed in their own lead shield with only the target leg exposed (Figure 1). Thus, the protocol was carried out twice to accommodate both the treatment and control groups. Each of the treatment and control mice was exposed to 5 Grays (Gy) of fractionated radiation at a dose of 1 Gray/min for 5 minutes. This protocol was repeated for 10 consecutive days for a total of 50 Grays of radiation exposure. Analysis was conducted throughout the radiation exposure period and beyond, as described below, and all mice were sacrificed at six weeks after their first exposure for further examination of histological changes between the treated and control populations. This time point was selected in order to capture long-term changes in the tissues, while limiting unnecessary animal stress.

### 2.5. Examination of Gross Appearance

All mice were housed individually and monitored daily for hair loss, skin ulceration, or other signs of tissue breakdown or general distress. Photographs of the left hind leg were taken before radiation exposure began and on every other subsequent day after for a total of six weeks.

### 2.6. Establishment of Functional Changes via Gait Analysis

TreadScan® 2.0 software (Clever Sys, Inc., Reston, VA) was used to monitor changes in physical performance due to the serial radiation exposure. Calibration of the system was achieved by running each mouse at 9 cm/sec and 15 cm/sec on the clear belt treadmill. Establishment of baseline performance was determined prior to radiation exposure by measuring each mouse's average run speed for the first one minute (instant run speed) and their average run speed for minutes two through five (overall run speed). After a rest period, each mouse repeated this exercise. All speeds were measured and recorded to the nearest mm/sec. This complete process was repeated on day 10, after the total radiation exposure had been received to determine if there was any change in the functional status of the treated or control mice. Averages and standard deviations were calculated for the two populations at both time points. Student's *t*-test was used to compare the groups and *p* ≤ 0.05 demonstrated a significant result.

### 2.7. Determination of Histological Changes

Postmortem tissue samples from the left quadriceps muscle of each of the treated and control mice were collected, stored in 10% formalin, and sent for paraffin embedding, sectioning, and staining. Samples were stained for the presence of survivin, to demonstrate the success of the gene-modification, and given an H&E stain, to determine the integrity of the tissue at the cellular level, and Masson's Trichrome stain, to highlight any fibrotic changes.

Staining for the presence of survivin was accomplished by fixing the slides and blocking them using serum-free protein block for 10 minutes. After washing, the primary antibody, survivin rabbit mAb (71G4B7, Cell Signaling, Beverly, Massachusetts), was applied at a concentration of 1 : 50 in Dako antibody diluent for 30 minutes and washed again. The secondary antibody (biotinylated goat anti-rabbit, Vector, Burlingame, California) was applied at 1 : 200 in protein block for 30 minutes and the final stain was visualized using DAB (Dako, Carpinteria, Calif). H&E and Masson's Trichrome staining were accomplished using standard laboratory techniques.

## 3. Results

### 3.1. Survivin Expression Helps Prevent Ulceration of the Skin and Improves Wound Healing following a Standard Radiation Therapy Protocol

**Figure 2:**
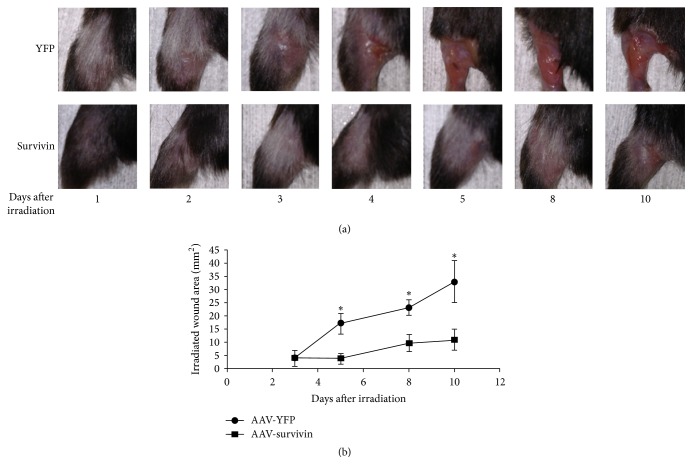
Mice treated with rAAV-survivin showed fewer signs of damage to the skin and surrounding tissue following 50 Gy of radiation exposure. Mice were monitored daily following the ten-day radiation protocol (a). Both YFP and survivin treated mice initially showed hair loss; however, by five days after the last dosage, there was a significant difference between the two groups in terms of the area of skin ulceration and impaired wound healing (b). Student's *t*-test was used to compare the groups and *p* ≤ 0.05 demonstrated a significant result. Data are mean ± s.d.; *n* = 5 per group. Student's *t*-test: *p* < 0.01 at five, eight, and ten days after radiation exposure. *∗* indicates significance.

Following gene-modification of thigh muscles to express survivin or control protein (YFP), mice were exposed to isolated radiation using the lead jig shown in Figure 1. Mice were visually inspected daily for changes in hair and skin quality secondary to radiation exposure (Figure 2(a)). Both the control (rAAV-YFP) and treated (rAAV-survivin) mice initially showed moderate hair loss over the dorsal aspect of the left hind limb. However, by five days after the last exposure, there was a significant difference between the two groups in terms of the area of skin ulceration and the impairment in wound healing, with the survivin treated population demonstrating a far more benign response to the radiation exposure (*p* < 0.01 at five, eight, and ten days after radiation exposure) (Figure 2(b)).

### 3.2. Compared to the rAAV-YFP Treated Mice, Mice Treated with rAAV-Survivin Performed Significantly Better on Physical Function Tests following Radiation Treatment

**Figure 3:**
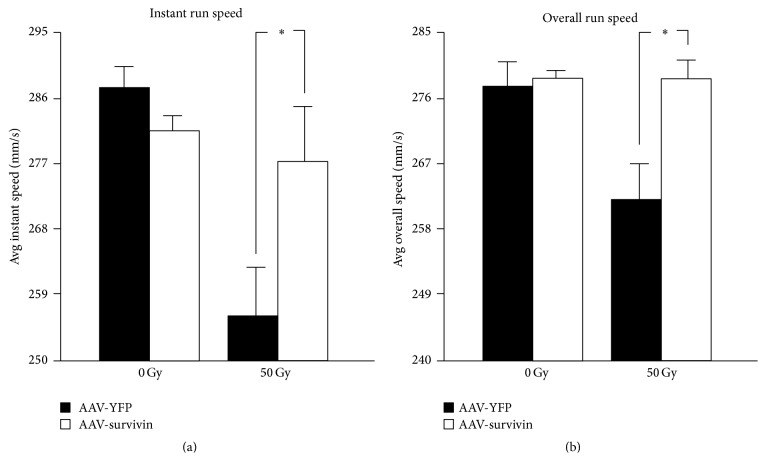
TreadScan software was used to track functional ability. Prior to beginning the radiation protocol, both groups of mice performed similarly on the treadmill. However, following 50 Gy of radiation, YFP mice showed a significant decrease in (a) instant run speed and (b) overall speed, while performance of survivin mice remained relatively unaffected. Student's *t*-test was used to compare the groups and *p* ≤ 0.05 demonstrated a significant result. Data are mean ± s.d.; *n* = 10 per group. Student's *t*-test: *p*_InstantRunSpeed_ **=** 0.05, *p*_OverallRunSpeed_ **=** 0.01. *∗* indicates significance.

TreadScan gait analysis software was used to determine baseline physical performance and any change after radiation exposure. Prior to beginning the radiation protocol, both groups of mice performed similarly on the treadmill. However, following 50 Gy of radiation, control mice showed a significant decrease in instant run speed (*p* = 0.05) and overall speed (*p* = 0.01), while performance of survivin mice remained relatively unaffected (Figures 3(a) and 3(b)).

### 3.3. Survivin Expression Mitigates the Negative Effects of Radiation Damage at the Cellular Level

**Figure 4:**
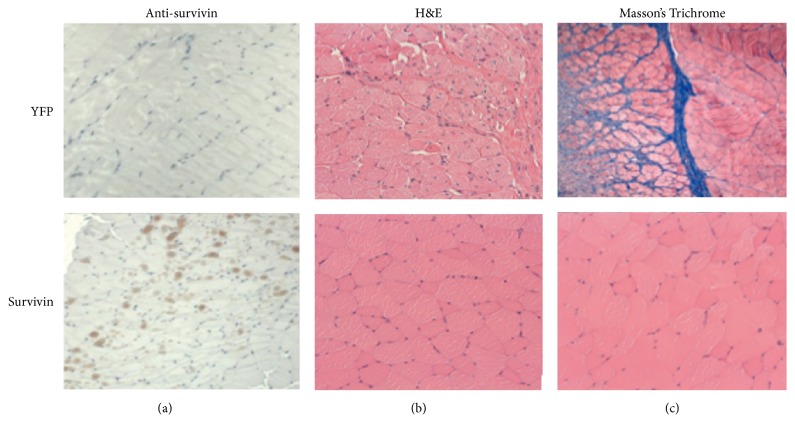
Histological analysis shows that survivin expression can mitigate the damaging effects of radiation therapy at the cellular level. After six weeks, mice were euthanized and muscle as well as the overlying dermal tissue at the site of radiation treatment was excised, stained, and examined. Mice treated with rAAV-survivin showed increased expression of the survivin protein (a). H&E staining showed that the integrity of the tissue was significantly affected at the cellular level in YFP mice but not in survivin expressing mice (b). Additionally, fibrosis was evident in the YFP mice but not in the survivin mice, as shown by staining with Masson's Trichrome (c).

Six weeks into the experiment, mice were euthanized and muscle along with overlying dermal tissue at the site of radiation treatment was excised, stained, and examined. Mice treated with rAAV-survivin showed the expected increase in expression of the survivin protein (Figure 4(a)). H&E staining demonstrated that the integrity of the tissue was significantly affected at the cellular level in YFP mice but not in survivin expressing mice. Additionally, histological analysis showed that the rAAV-YFP treated mice had substantial thickening of the dermal tissue, as well as an increase in collagen that was not present in the survivin treated mice (Figure 4(b)). Finally, fibrosis was evident only in the YFP mouse samples, as shown by staining with Masson's Trichrome (Figure 4(c)).

## 4. Discussion

For many orthopedic oncologic cases, adjuvant radiation therapy may effectively eradicate residual cancer cells from the tumor resection bed. However, ionizing radiation is not specific for only cancer cells and frequently causes damage to normal surrounding tissues as well. It has been reported that soft tissue complications occur in up to 60% of patients following radiation therapy [6]. These complications can include atrophy, fibrosis, desquamation, and ulceration [13]. These processes are especially harmful in the postoperative setting, when tissue viability is essential for proper healing of the reconstruction and for maintenance of a soft tissue barrier to prevent wound infection. It has been shown that repeated radiation exposure leads to upregulation and overexpression of growth factors, including tumor growth factor beta (TGF-b), fibroblast growth factor (FGF), and vascular endothelial growth factor (VEGF), which results in the formation of the fibrotic changes that are typically seen in the irradiated patient [6].

In our study, we report the use of gene-modification techniques to induce survivin expression in normal tissues to allow for the protection of nontarget areas from the negative side effects normally associated with ionizing radiation. Following radiation exposure, the control group demonstrated significant ulceration of the irradiated area while the survivin treated mice exhibited only moderate hair loss. Additionally, histological analysis showed that the control mice experienced a significant thickening of the dermal tissue, as well as an increase in collagen that was not present in the survivin treated mice. Finally, gait analysis demonstrated a significant difference in the instant run speed and overall run speed between the treated and control groups, with the control mice averaging a significantly lower speed in both cases.

In an effort to reduce or even prevent the adverse effects on soft tissue associated with radiation therapy, researchers have long been exploring techniques to promote the repair of tissues which have already been injured by radiation [13, 14, 27]. One of the most classic protocols for the treatment of radiation-induced damage employs the use of hyperbaric oxygen. This method has been frequently used to promote wound healing after a wide range of tissue injuries and multiple studies have shown that the increase in oxygen pressure leads to increased neovascularization and more rapid production of a high quality scar [19]. While unquestionably useful, more recent research has focused on not just treating the injured tissues but actually preventing them.

A 2013 meta-analysis by Zhang et al. compiled all of the previously published reports of topically applied compounds designed to prevent soft tissue damage caused by radiation [14]. The authors cited 14 studies which promoted the use of a wide range of products, from beclomethasone to* Aloe vera*, for radiation protection [17, 18]. While individual studies suggested that these may be reasonable options for preventing wound breakdown, no one agent proved to be successful across multiple studies in doing more than providing tissue moisturization or minor anti-inflammatory effects. Additionally, these products were limited by the need to reapply them prior to every radiation exposure. Ultimately, a more dramatic improvement in wound healing would most likely require protocols that induce changes at the molecular level and can be sustained throughout the entire course of radiation treatment.

The goal of our study was to design a gene therapy protocol to introduce the mitosis regulating protein, survivin, to nontarget tissues in the radiation field in an attempt to confer radiation resistance to normal, healthy cells. Adenoassociated viruses (AAV), which have consistently been the choice for gene therapy regimens due to their inability to transmit human disease, were modified to create unique recombinant AAV vectors (rAAV) which have proven to exhibit enhanced tissue tropism [28, 29]. The novel rAAV, rAAVrec2, which is specific to our laboratory has been employed in our previously published experiments which demonstrated its advantages over traditional viral serotypes [24, 25]. Using rAAVrec2, we were able to successfully localize the expression of survivin to the targeted radiation area, while preventing any systemic effects.

Survivin is a known inhibitor of apoptosis (IAP) and has been found in high levels in many cancer cell lines, as well as in the developing human fetus. Although the details of survivin's mechanism of action are still unclear, it has been well established that the loss of this protein leads to cell death during interphase and that upregulation leads to cellular proliferation [21, 22]. Therefore, survivin is the ideal protein for preventing unwanted cell death in normal tissues following radiation exposure. Our experiments indicate that using gene therapy techniques to locally introduce survivin to the normal tissues prior to radiation exposure dramatically reduces ulceration and fibrotic changes seen when radiation therapy is given alone.

While our initial experiments did show promising results, future studies are still needed to determine exactly what cells are affected by the introduction of survivin and whether it is possible to safely modify healthy tissue without conferring radiation resistance to any residual tumor cells. Additionally, success of the protocol in a murine model does not necessarily imply success in a larger animal model. Porcine experiments are necessary to verify the feasibility of our gene-modification technique on a larger scale before translation to humans could even be considered. Finally, data regarding the duration of survivin expression over a longer period of time is also needed. It is possible that gene-modification would have to be repeated midway through radiation treatment if the regimen was particularly long or if the territory of the irradiated area was extremely large. These are all issues that warrant further exploration.

## 5. Conclusions

Using gene-modification techniques, we have been able to introduce survivin expression into normal tissues and induce the protection of nontarget soft tissues from the damaging effects of ionizing radiation. This could potentially reduce the need for reoperation due to wound breakdown after oncological resection and allow for improved wound healing after reconstruction. Specifically in cases, such as Ewing sarcoma, where the rate of local recurrence is high and the need for extended radiation is great, gene-modification with survivin could prove invaluable for a successful postoperative recovery.
